# Derived Neutrophils to Lymphocyte Ratio Predicts Survival Benefit from TPF Induction Chemotherapy in Local Advanced Oral Squamous Cellular Carcinoma

**DOI:** 10.3390/cancers16152707

**Published:** 2024-07-30

**Authors:** Fangxing Zhu, Xinyu Zhou, Yiyi Zhang, Zhihang Zhou, Yingying Huang, Laiping Zhong, Tongchao Zhao, Wenjun Yang

**Affiliations:** 1 Department of Oral & Maxillofacial-Head & Neck Oncology, Shanghai Ninth People’s Hospital, Shanghai Jiao Tong University School of Medicine, No. 639, Zhizaoju Road, Shanghai 200011, China; sp_terry94@163.com (F.Z.); drzhouxy98@126.com (X.Z.); nanamissx@163.com (Y.Z.); zhihangz@126.com (Z.Z.); kqyxyhyy@163.com (Y.H.); 2 College of Stomatology, Shanghai Jiao Tong University, No. 639, Zhizaoju Road, Shanghai 200011, China; 3 National Center for Stomatology, Shanghai 200011, China; 4 National Clinical Research Center for Oral Diseases, No. 639, Zhizaoju Road, Shanghai 200011, China; 5 Shanghai Key Laboratory of Stomatology, Shanghai 200011, China; 6 Shanghai Research Institute of Stomatology, No. 639, Zhizaoju Road, Shanghai 200011, China; 7 Department of Stomatology, Huashan Hospital, Fudan University, Shanghai 200040, China; zhonglaiping9th@163.com; 8 Huangpu Branch, Shanghai Ninth People’s Hospital, Shanghai JiaoTong University School of Medicine, No. 58, Pu Yu Dong Road, Shanghai 200011, China

**Keywords:** dNLR, oral cancer, prognosis, TPF, induction chemotherapy

## Abstract

**Simple Summary:**

This study aimed to evaluate the derived neutrophil to lymphocyte ratio (dNLR) in predicting the prognosis of patients with locally advanced oral squamous cell carcinoma, as well as the survival benefits from induction chemotherapy with docetaxel, cisplatin, and 5-fluorouracil (5-FU). The dNLR is an independent negative predictive factor for the disease. Patients with cTNM stage III disease and a low dNLR may benefit from induction chemotherapy.

**Abstract:**

Background: This study aimed to evaluate the derived neutrophil to lymphocyte ratio (dNLR) in predicting the prognosis of patients with locally advanced oral squamous cell carcinoma (LAOSCC) and to assess the survival benefits from docetaxel, cisplatin, and 5-fluorouracil (5-FU) (TPF) induction chemotherapy (IC). Methods: Patients from a phase III trial involving TPF IC in stage III/IVA OSCC patients (NCT01542931) were enrolled. Receiver operating characteristic curves were constructed, and the area under the curve was computed to determine dNLR cutoff points. Kaplan–Meier survival estimates and Cox proportional hazards models were used for longitudinal analysis. Results: A total of 224 patients were identified (median age: 55.4 years; range: 26 to 75 years; median follow-up: 90 months; range: 3.2 to 93 months). The cutoff point for the dNLR was 1.555. Multivariate analysis showed that the dNLR was an independent negative predictive factor for survival (overall survival (OS): hazard ratio (HR) = 1.154, 95% confidence interval (CI): 1.018–1.309, *p* = 0.025; disease-free survival (DFS): HR = 1.123, 95% CI: 1.000–1.260, *p* = 0.050; local recurrence-free survival (LRFS): HR = 1.134, 95% CI: 1.002–1.283, *p* = 0.047; distant metastasis-free survival (DMFS): HR = 1.146, 95% CI: 1.010–1.300, *p* = 0.035). A low dNLR combined with cTNM stage III disease predicted benefit from TPF IC for the patients [OS (χ^2^ = 4.674, *p* = 0.031), DFS (χ^2^ = 7.134, *p* = 0.008), LRFS (χ^2^ = 5.937, *p* = 0.015), and DMFS (χ^2^ = 4.832, *p* = 0.028)]. Conclusions: The dNLR is an independent negative predictive factor in LAOSCC patients. Patients with cTNM stage III disease and a low dNLR can benefit from TPF IC.

## 1. Introduction

Oral squamous cell carcinoma (OSCC) is the most common tumor in the oral and maxillofacial region, accounting for 90% of lip and oral cavity cancer [1] and ranking as the 16th most common malignancy worldwide [2]. The global annual estimated incidence of lip and oral cavity cancer was 389,485 incident cases in 2022 [2]. According to the National Comprehensive Cancer Network (NCCN) Clinical Practice Guidelines Version 1.2022 [3], the mainstream management of OSCC remains radical surgery combined with sequential therapy. This approach, however, compromises patients’ quality of life due to the extensive excision and neck dissection, followed by postoperative radiation. Such treatments can impair critical functions of the oral and maxillofacial region, including eating, drinking, swallowing, and speaking, as well as alter appearance, posing significant threats to patients’ lives. In most countries, the five-year survival rates for cancers of the tongue, oral cavity, and oropharynx are around 50% [4], with a total of 188,230 deaths from cancer of the lip and oral cavity reported in 2022 [2]. Therefore, it is urgent to identify the most effective treatment strategy for the appropriate patients.

Although various neoadjuvant therapies have been tested in clinical trials, challenging the traditional standard therapy (radical surgery and postoperative radiation), no single treatment strategy has been universally beneficial. In our previous phase III clinical trial, docetaxel, cisplatin, and 5-fluorouracil (5-FU) (TPF) were used as induction chemotherapy in patients with locally advanced oral squamous cell carcinoma (LAOSCC) (registration ID: NCT01542931). However, only a portion of pathologic responders benefited from this approach [5]. Other phase III clinical trials have shown varying survival benefits from additional TPF induction chemotherapy [6,7,8]. In the NCT00095875 trial, TPF induction chemotherapy did not provide extra survival benefits beyond concurrent chemoradiotherapy in locally advanced head and neck cancer [6]. Conversely, the NCT01245959 trial demonstrated that adding TPF induction chemotherapy to concurrent chemoradiotherapy significantly improved failure-free survival in locoregionally advanced nasopharyngeal carcinoma with acceptable toxicity [7]. Similarly, the NCT01086826 trial showed that TPF induction chemotherapy significantly improved radiological complete responses, progression-free survival, and overall survival without compromising compliance with concomitant platinum-based chemoradiotherapy in locally advanced head and neck squamous cell carcinoma [8]. These findings highlight the potential value of neoadjuvant therapy, suggesting that only a subset of patients benefit from TPF induction chemotherapy [5]. This underscores the need for a personalized treatment strategy to identify specific patients who will benefit. The rise of personalized medicine and the increasing importance of biomarkers in tailoring treatment strategies have led to numerous clinical trials exploring different approaches. In our previous studies, growth differentiation factor 15 (GDF15) was identified as a potential predictive biomarker, with patients exhibiting cN- and high expression of GDF15 benefiting from TPF induction chemotherapy in LAOSCC [9]. Additionally, normal body mass index (BMI) was found to predict survival benefits from TPF induction chemotherapy in patients with stage IVA cancer within the same cohort [10].

The derived neutrophil to lymphocyte ratio (dNLR) is an index easily obtained from a routine complete blood count (CBC) test. It is calculated as the ratio of neutrophils to the difference between leukocytes and neutrophils, reflecting the relative quantity of tumor-immune-related cells in leukocytes. A smaller dNLR indicates more tumor-immune-related cell infiltration [11]. The prognostic value of the dNLR has been demonstrated in various cancers, including pancreatic ductal adenocarcinoma [12], breast cancer [13], non-small cell lung cancer [14,15], advanced or metastatic melanoma [16,17], metastatic prostate cancer [18,19], metastatic renal cell carcinoma [20], advanced or metastatic colorectal cancer [21,22], intrahepatic cholangiocarcinoma [15], and upper tract urothelial carcinoma [23]. However, in the field of head and neck squamous cell carcinoma (HNSCC), there is no clear evidence supporting the dNLR as a prognostic biomarker [24,25]. It has been suggested that the slightly decreased prognostic value might be due to a smaller area under the curve-receiver operating characteristic (AUC-ROC) for predicting overall survival (OS) [24]. Additionally, no literature has reported on the relationship between the dNLR and survival benefits from TPF induction chemotherapy. Despite this, TPF induction chemotherapy has shown benefits in previous trials (NCT01542931, NCT01245959, and NCT01086826) [5,7,8]. Therefore, it is crucial to identify the exact patient population that will benefit from TPF induction chemotherapy. In summary, this study aimed to demonstrate our findings on the predictive value of the dNLR for survival benefits from TPF induction chemotherapy in patients with LAOSCC.

## 2. Patients and Methods

### 2.1. Patients

OSCC patients diagnosed with TNM clinical stage III or IVA, treated in the Department of Oral and Maxillofacial-Head and Neck Oncology at Shanghai Ninth People’s Hospital, Shanghai Jiao Tong University School of Medicine, Shanghai, China, were enrolled in this study. The cohort participated in a prospective, randomized, phase III trial (NCT01542931) to investigate the potential benefits of TPF induction chemotherapy [5]. Patients were randomly allocated to either the experimental group (TPF induction chemotherapy, surgery, and postoperative radiotherapy) or the control group (surgery and postoperative radiotherapy). Clinical data from the control group were used to evaluate the prognostic value of the following biomarkers: derived neutrophil to lymphocyte ratio (dNLR), neutrophil to lymphocyte ratio (NLR), platelet to lymphocyte ratio (PLR), lymphocyte to monocyte ratio (LMR), and pan-immune inflammation value (PIV).

### 2.2. Ethical Approval

The study was conducted in accordance with the Declaration of Helsinki (as revised in 2013) and approved by the Shanghai Ninth People’s Hospital institutional review board. Signed informed consent forms were obtained from all patients.

### 2.3. Baseline Characteristics

All clinical baseline characteristics and data were measured and recorded when patients were first referred to our department. This information was extracted from electronic medical records, independently recorded, and verified by the researchers. The biomarkers were calculated from the baseline CBC. dNLR is defined as the absolute neutrophil count divided by the white cell count minus the absolute neutrophil count; NLR is defined as the absolute neutrophil count divided by the absolute lymphocyte count; PLR is defined as the platelet count divided by the absolute lymphocyte count; LMR is defined as the absolute lymphocyte count divided by the absolute monocyte count; and PIV is defined as the absolute neutrophil count multiplied by the platelet count multiplied by the absolute monocyte count divided by the absolute lymphocyte count. Positive alcohol use and smoking status were defined as previously described [5].

### 2.4. TPF Induction Chemotherapy and Standard Treatment

The treatment strategy was reported previously [5]. In brief, the palpable edges of the primary lesion (both the longest and shortest axes) were marked 0.5 cm away from the lesion before induction chemotherapy. Chemotherapy consisted of docetaxel 75 mg/m^2^ intravenously on day 1, followed by cisplatin 75 mg/m^2^ intravenously on day 1, and fluorouracil 750 mg/m^2^ per day as a 120-hour continuous intravenous infusion on days 1 through 5. TPF induction chemotherapy was administered every 3 weeks for two cycles, unless there was disease progression or unacceptable toxicity. For the surgical component, radical resection of the primary lesion and full neck dissection (functional or radical) with appropriate reconstruction (pedicle or free flap) were performed. Radiotherapy was initiated 4 to 6 weeks after surgery. Standard conformal or intensity-modulated radiotherapy was administered at a dose of 1.8 to 2 Gy per day, 5 days per week, for 6 weeks (totaling 54 to 60 Gy). No concurrent chemotherapy was given with postoperative radiotherapy.

### 2.5. Follow-Up Visit and Clinical End-Point Assessment

Patients were followed every three months for the first two years, every six months for the next three to five years, and once a year thereafter until death or data censorship. The primary outcome of this study was overall survival (OS), calculated from the day of random assignment to death. The secondary outcomes included disease-free survival (DFS), locoregional recurrence-free survival (LRFS), and distant metastasis-free survival (DMFS), calculated from the day of random assignment to cancer recurrence, locoregional recurrence, distant metastasis, or death from any cause.

### 2.6. Statistical Analysis

Continuous variables were presented as the mean ± SD. For comparing clinical and histologic data, χ^2^ tests were used for categorical variables, and the Student’s t-test or Mann–Whitney U test were used for continuous variables. ROC curves were constructed, and AUC values were computed. The Youden index was used to determine the optimal cutoff points for the dNLR. A prognostic multivariate model was built using Cox regression analysis to investigate the hazard ratio (HR). To validate the assumptions of the Cox proportional hazards model, we tested for the proportionality of hazards using Schoenfeld residuals. The Schoenfeld residuals were plotted and statistically assessed to ensure no significant deviations from the proportional hazards assumption. Additionally, model fit was evaluated using the Akaike Information Criterion (AIC) to ensure the robustness and reliability of our results, as previously indicated [26,27]. The variables included in the multivariate model were determined by univariate Cox regression analysis and clinical evaluation. The Kaplan–Meier method and log-rank test were used for survival analysis. Two-sided tests with a significance level of 0.05 were adopted for all hypothesis-generating tests. The data were analyzed using IBM SPSS Statistics 25, IBM Corporation (Armonk, NY, USA).

## 3. Results

### 3.1. Patients

There were 256 patients [179 (69.9%) males and 77 (30.1%) females, with an average age of 55.4 years (range 26 to 75 years), and 168 (66.4%) patients older than 60 years] enrolled in the previous trial. Of these, 224 patients completed the treatment, 29 patients did not complete the treatment, and 3 patients declined any treatment. In the experimental group (TPF induction chemotherapy, surgery, and postoperative radiotherapy), there were 109 patients who completed the treatment. In the control group (surgery and postoperative radiotherapy), there were 115 patients who completed the treatment (including 2 patients who declined TPF induction chemotherapy). The median follow-up time was 80 months (range: 3.2 to 93 months).

Regarding the dNLR, the mean value in the control group was 1.89 ± 1.45, while in the TPF chemotherapy induction group, it was 1.81 ± 1.59. There was no statistically significant difference between the two groups in the baseline dNLR (U = 7411, *p* = 0.187). The most common primary tumor site was the tongue (113 [44.1%]), followed by the buccal mucosa (45 [17.6%]), gingiva (39 [15.6%]), floor of the oral cavity (30 [11.7%]), palate (18 [7.0%]), and the retromolar trigone (10 [3.9%]). The clinical TNM stages were distributed as follows: III (177 [69.1%]) and IVA (79 [30.9%]). Detailed clinical characteristics and treatments are shown in Table 1.

### 3.2. The dNLR Predicts Survival Outcomes in LAOSCC Patients Treated by Surgery and Postoperative Radiation (the Control Group)

**Figure 1:**
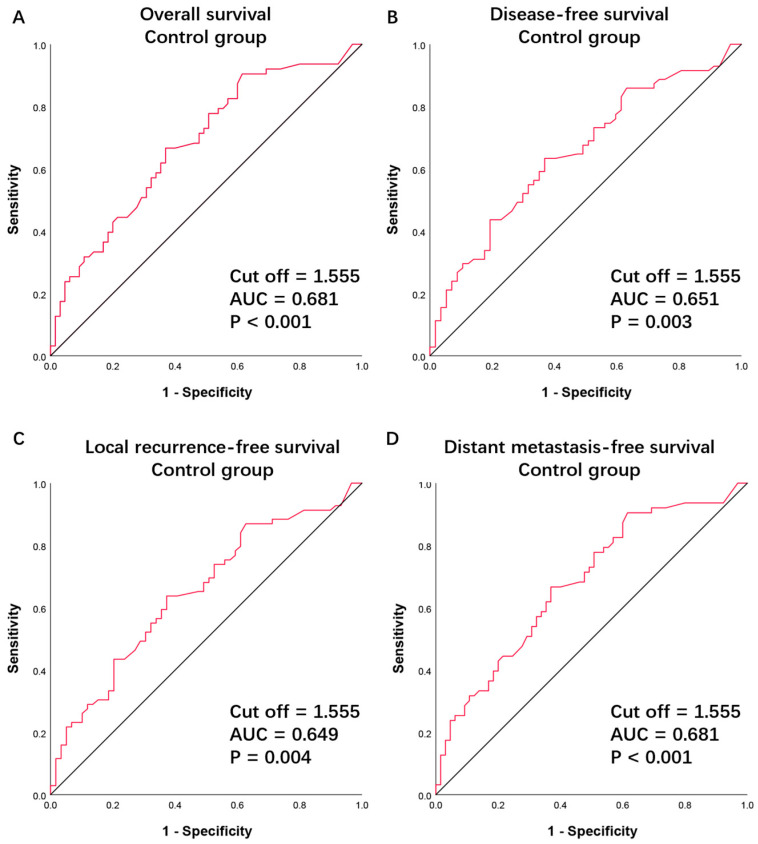
Receiver operating characteristic curve for (**A**) overall survival, (**B**) disease-free survival, (**C**) locoregional recurrence-free survival, and (**D**) distant metastasis-free survival in patients treated with surgery and postoperative radiation (the control group). AUC, area under the curve.

According to the baseline CBC, the continuous variable dNLR had an average value of 1.89, ranging from 0.20 to 10.75. A cutoff point of 1.555 was identified to predict OS, calculated based on the ROC curves and the Youden’s index (*p* < 0.001, AUC = 0.681, Figure 1A, Supplementary Table S1). This same cutoff point was confirmed for DFS (*p* = 0.003, AUC = 0.651, Figure 1B, Supplementary Table S2), LRFS (*p* = 0.004, AUC = 0.649, Figure 1C, Supplementary Table S3), and DMFS (*p* < 0.001, AUC = 0.681, Figure 1D, Supplementary Table S4). Subsequently, patients in both the control and experimental groups were categorized into high dNLR (>1.555) and low dNLR (≤1.555) groups. In the control group, 66 patients were allocated to the high dNLR group and 62 to the low dNLR group. In the experimental group, 73 patients were allocated to the high dNLR group and 55 to the low dNLR group. It was found that in the control group, there was a statistical correlation between clinical stage and dNLR, with stage IVA disease being more associated with a high dNLR (χ^2^ = 3.863, *p* = 0.049, Table 1).

**Figure 2:**
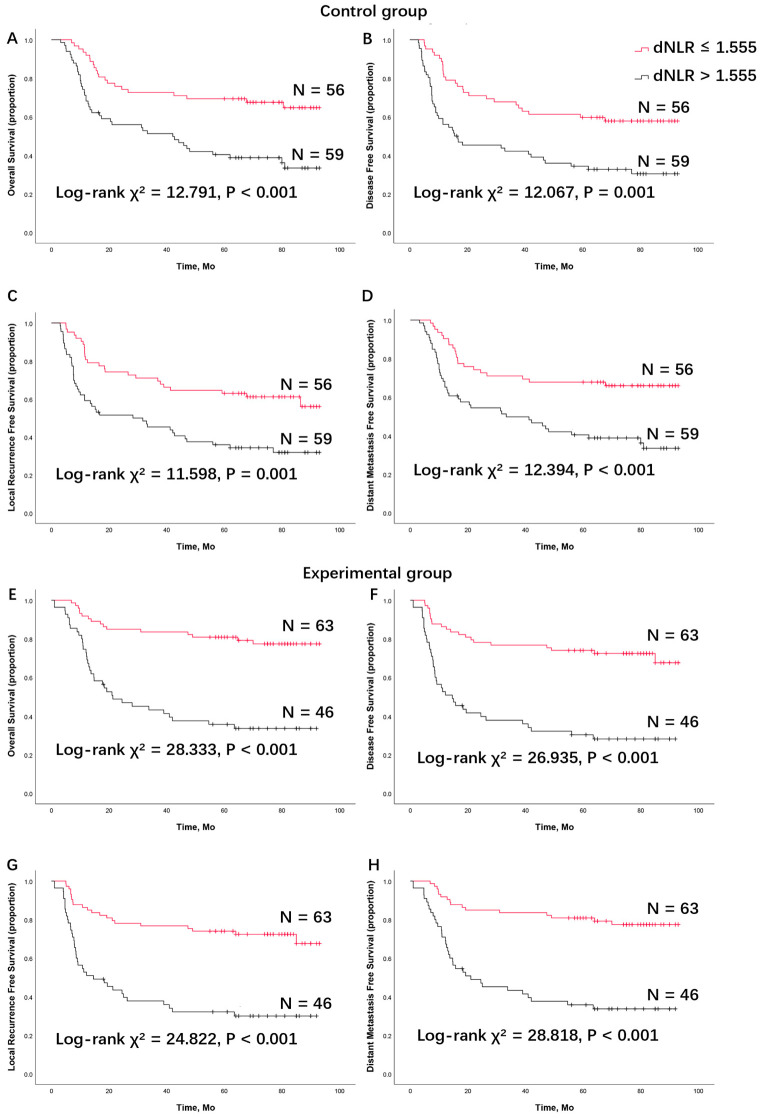
Survival analysis of (**A**) overall survival, (**B**) disease-free survival, (**C**) locoregional recurrence-free survival, and (**D**) distant metastasis-free survival between patients with a low derived neutrophils to lymphocyte ratio and a high derived neutrophils to lymphocyte ratio in patients treated with surgery and postoperative radiation (the control group). Survival analysis of (**E**) overall survival, (**F**) disease-free survival, (**G**) locoregional recurrence-free survival, and (**H**) distant metastasis-free survival in patients treated with TPF induction chemotherapy, surgery, and postoperative radiation (the experimental group). dNLR, derived neutrophils to lymphocyte ratio; TPF, docetaxel, cisplatin, and 5-fluorouracil (5-FU).

To further verify the effectiveness of the dNLR cutoff point of ≤1.555 in predicting a good prognosis in the control group, survival analysis was performed, comparing patients with a high dNLR and a low dNLR. The Kaplan–Meier analysis showed that patients with a low dNLR had significantly longer OS, DFS, LRFS, and DMFS compared to patients with a high dNLR. According to the log-rank test, patients with a low dNLR had significantly longer OS (χ^2^ = 12.791, *p* < 0.001), DFS (χ^2^ = 12.067, *p* = 0.001), LRFS (χ^2^ = 11.598, *p* = 0.001), and DMFS (χ^2^ = 12.394, *p* < 0.001) compared to those with a high dNLR in the control group (Figure 2). In the control group, patients with a high dNLR had a lower 5-year survival rate. The 5-year survival rates for OS, DFS, LRFS, and DMFS were 40.4%, 34.4%, 35.9%, and 40.4%, respectively, for high dNLR patients, whereas the rates were 69.4%, 59.7%, 62.9%, and 67.7%, respectively, for low dNLR patients (Table 2).

Univariate Cox model analyses were conducted in the control group. The BMI at diagnosis, cTNM stage (vs. stage III), and dNLR were found to have significant correlations with OS, DFS, LRFS, and DMFS (Table 3). The underweight group had worse survival outcomes compared to the normoweight group (OS: HR = 2.145, 95% CI: 1.065–4.321, *p* = 0.033; LRFS: HR = 2.106, 95% CI: 1.053–4.210, *p* = 0.035; DMFS: HR = 2.067, 95% CI: 1.027–4.162, *p* = 0.042). The overweight and obese groups had better clinical survival outcomes than the normoweight group, but this did not show statistical significance. Additionally, the cTNM stage showed statistical significance with survival outcomes (stage IVA vs. III: OS: HR = 1.993, 95% CI: 1.184–3.354, *p* = 0.009; DFS: HR = 1.792, 95% CI: 1.088–2.952, *p* = 0.022; LRFS: HR = 1.939, 95% CI: 1.173–3.207, *p* = 0.010; DMFS: HR = 1.603, 95% CI: 1.127–3.190, *p* = 0.016). In terms of the dNLR, statistically significant differences were found in survival outcomes (OS: HR = 1.227, 95% CI: 1.099–1.369, *p* < 0.001; DFS: HR = 1.186, 95% CI: 1.063–1.324, *p* = 0.002; LRFS: HR = 1.200, 95% CI: 1.073–1.341, *p* = 0.001; DMFS: HR = 1.218, 95% CI: 1.091–1.361, *p* < 0.001). The PLR also showed slightly but statistically significant differences in survival outcomes (DFS: HR = 1.004, 95% CI: 1.000–1.008, *p* = 0.044; LRFS: HR = 1.005, 95% CI: 1.000–1.009, *p* = 0.030). The proportionality of hazards was assessed using Schoenfeld residuals, which confirmed no significant deviations from the proportional hazards assumption (Supplementary Figure S4). The model fit was evaluated using the Akaike Information Criterion (AIC), and the results indicated a well-fitting model with AIC values supporting the robustness of the Cox regression analyses performed (Supplementary Table S5).

To investigate the applicability of the aforementioned prognostic factors, a multivariate analysis was adjusted for potentially confounding clinical variables. Both the cTNM stage (stage IVA vs. III, OS: HR = 1.911, 95% CI: 1.098–3.328, *p* = 0.022; LRFS: HR = 1.756, 95% CI: 1.029–2.996, *p* = 0.039; DMFS: HR = 1.797, 95% CI: 1.003–3.125, *p* = 0.038) and the dNLR (OS: HR = 1.154, 95% CI: 1.018–1.309, *p* = 0.025; DFS: HR = 1.123, 95% CI: 1.260–1.000, *p* = 0.050; LRFS: HR = 1.134, 95% CI: 1.002–1.283, *p* = 0.047; DMFS: HR = 1.146, 95% CI: 1.010–1.300, *p* = 0.035) were found to be independent predictive factors in the control group (Table 4). The PLR showed no statistical significance in the multivariate analysis.

### 3.3. The dNLR Predicts Survival Outcomes in LAOSCC Patients Treated by TPF Induction Chemotherapy, Surgery, and Postoperative Radiation (the Experimental Group)

In the experimental group, patients were further allocated into two subgroups based on their baseline dNLR, using the cutoff point of 1.555 from the control group. The five-year OS, DFS, LRFS, and DMFS were 35.7%, 30.3%, 32.1%, and 35.7%, respectively, in patients with a high dNLR, compared to 80.8%, 74.0%, 74.0%, and 80.8%, respectively, in the low dNLR group (Table 2). According to the log-rank test, patients in the low dNLR group had significantly longer OS (χ^2^ = 28.333, *p* < 0.001), DFS (χ^2^ = 26.935, *p* < 0.001), LRFS (χ^2^ = 24.822, *p* < 0.001), and DMFS (χ^2^ = 28.818, *p* < 0.001).

Univariate Cox model analyses revealed that the cTNM stage (stage IVA vs. III, OS: HR = 1.588, 95% CI: 1.015–2.755, *p* = 0.022; DFS: HR = 2.461, 95% CI: 1.398–4.331, *p* = 0.002; LRFS: HR = 2.493, 95% CI: 1.413–4.399, *p* = 0.009; DMFS: HR = 1.603, 95% CI: 1.060–3.500, *p* = 0.031), the dNLR (OS: HR = 1.154, 95% CI: 1.035–1.285, *p* = 0.010; DFS: HR = 1.141, 95% CI: 1.029–1.266, *p* = 0.013; LRFS: HR = 1.139, 95% CI: 1.024–1.268, *p* = 0.016; DMFS: HR = 1.152, 95% CI: 1.034–1.282, *p* = 0.010), the NLR (DFS: HR = 1.326, 95% CI:1.015–1.731, *p* = 0.038; LRFS: HR = 1.315, 95% CI: 1.005–1.722, *p* = 0.046), and the PLR (DFS: HR = 1.009, 95% CI: 1.002–1.017, *p* = 0.017; LRFS: HR = 1.008, 95% CI: 1.000–1.016, *p* = 0.040) had statistically significant correlations with the survival outcomes (Table 5).

A multivariate Cox model analysis further confirmed that the cTNM stage was an independent predictive factor for some survival outcomes (OS: HR = 1.924, 95% CI: 1.057–3.499, *p* = 0.032; DFS: HR = 1.926, 95% CI: 1.154–3.215, *p* = 0.012; LRFS: HR = 1.991, 95% CI: 1.188–3.337, *p* = 0.024; DMFS: HR = 1.926, 95% CI: 1.014–2.782, *p* = 0.033). Additionally, the dNLR was identified as an independent predictive factor for all survival outcomes (OS: HR = 1.160, 95% CI: 1.031–1.276, *p* = 0.011; DFS: HR = 1.239, 95% CI: 1.019–1.250, *p* = 0.021; LRFS: HR = 1.125, 95% CI: 1.014–1.249, *p* = 0.027; DMFS: HR = 1.145, 95% CI: 1.030–1.272, *p* = 0.012) (Table 6).

### 3.4. Combining the cTNM Stage and dNLR to Predict the Benefit of TPF Induction Chemotherapy for LAOSCC Patients

**Figure 3:**
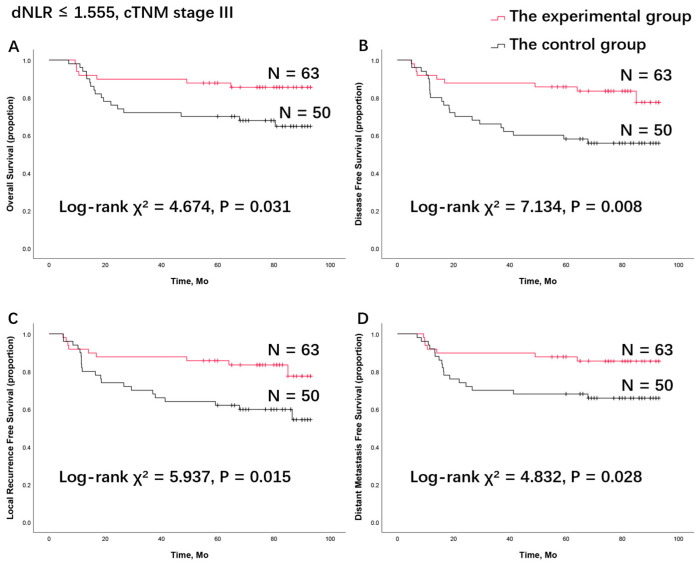
Survival analysis of (**A**) overall survival, (**B**) disease-free survival, (**C**) locoregional recurrence-free survival, and (**D**) distant metastasis-free survival in the patient group with a low dNLR combined with cTNM stage III disease between the control group and the experimental group. dNLR, derived neutrophils to lymphocyte ratio; TPF, docetaxel, cisplatin, and 5-fluorouracil (5-FU).

After grouping patients based on management methods (control group or experimental group) and combining the independent predictive factors previously verified, different subgroups were created according to the cTNM stage and dNLR level. The Kaplan–Meier method and log-rank test were used for survival analysis (Figure 3).

When comparing the survival rates of the experimental group and control group, as reported in our previous publications, no statistical significance was found between the two treatment groups (Supplement Figure S1). Surprisingly, in the subgroup of patients with cTNM stage III disease and a low dNLR (dNLR ≤ 1.555), the log-rank test indicated that patients in the experimental group had significantly longer OS (χ^2^ = 4.674, *p* = 0.031), DFS (χ^2^ = 7.134, *p* = 0.008), LRFS (χ^2^ = 5.937, *p* = 0.015), and DMFS (χ^2^ = 4.832, *p* = 0.028) compared to the control group (Figure 3). This suggests that patients with cTNM stage III disease and a low dNLR (dNLR ≤ 1.555) can benefit from TPF induction chemotherapy. In other subgroups, divided solely by cTNM stage or dNLR level, no statistically significant survival benefit from TPF induction chemotherapy was observed (Supplement Figures S2 and S3).

## 4. Discussion

In this study, the CBC, clinical characteristics, and survival outcomes of LAOSCC patients were retrospectively analyzed. In the control group, through univariate and multivariate Cox model analyses, the baseline dNLR (as a continuous variable) was confirmed as an independent prognostic factor for survival outcomes (Table 3 and Table 4). Using the ROC curve and Youden’s index (Figure 1), a dNLR cutoff point of 1.555 was established for all survival outcomes (OS, DFS, LRFS, and DMFS). Based on this cutoff point, patients were categorized into two subgroups: low dNLR and high dNLR. Patients with a low baseline dNLR had better survival outcomes (Figure 2), and this finding was consistent in the experimental group (Table 5 and Table 6). Concurrently, the cTNM stage (as a categorical variable) was confirmed as an independent prognostic factor for survival outcomes. When combining the two variables—cTNM stage III disease and low dNLR (dNLR ≤ 1.555)—this specific patient population showed significant benefit from TPF induction chemotherapy (Figure 3).

In recent years, many immune biomarkers easily acquired from a CBC have been introduced into the field of tumor therapy as prognostic factors. These include the neutrophil to lymphocyte ratio (NLR) [28,29,30], the derived neutrophil to lymphocyte ratio (dNLR) [11,14,18,31], the platelet to lymphocyte ratio (PLR) [32,33,34], the lymphocyte to monocyte ratio (LMR) [20,35], and the pan-immune inflammation value (PIV) [36,37]. In OSCC, the NLR has been reported as a significant independent predictor of disease-specific survival (DSS) [38,39] and is significantly correlated with stromal infiltration of CD8+, CD4+, and CD20+ lymphocytes [40]. The dNLR has been correlated with the occurrence of complications [41]. The PLR has shown a stronger association with DSS and progression-free survival (PFS) in patients who are male, have stage III/IV OSCC, or have lymph node metastasis [42]. The NLR and dNLR appear similar and have comparable effects on cancer-specific mortality [43], and a positive correlation between the dNLR and NLR has been found [44]. However, the calculation method for the dNLR includes not only lymphocytes but also monocytes and other subtypes of immune-related cells. In this study, dNLR, NLR, PLR, LMR, and PIV were analyzed using univariate and multivariate Cox regression analyses. Only the dNLR demonstrated an independent negative effect on survival prognosis (Table 3, Table 4, Table 5 and Table 6). We believe the complexity of the tumor immune microenvironment and the neutrophil heterogeneity among OSCC patients contribute to these results.

To our knowledge, this is the first time that the dNLR has been confirmed as an independent prognostic factor for the survival of OSCC patients through multivariate analysis. These patients were treated either with surgery and postoperative radiation or with TPF induction chemotherapy, surgery, and postoperative radiation. Additionally, this is the first time that the dNLR level and cTNM stage have been confirmed as criteria for identifying patient populations that can benefit from TPF induction chemotherapy (Figure 3). In our previous phase III clinical trial (registration ID: NCT01542931), TPF induction chemotherapy did not improve survival compared with upfront surgery [5]. However, patients with cN2 disease appeared to have improved OS (HR, 0.418; 95% CI, 0.179 to 0.974; *p* = 0.043) and DMFS (HR, 0.418; 95% CI, 0.179 to 0.974; *p* = 0.043) when treated with TPF induction chemotherapy compared to those who were not [5]. Thus, efforts were made to identify clinically valuable biomarkers to screen appropriate OSCC patient populations for TPF induction chemotherapy. Lymph node ratio (LNR), the ratio of pathologically confirmed positive lymph nodes to the total number of surgically removed lymph nodes, was found to be connected to prognosis and could be an independent prognostic factor. OSCC patients with high-risk LNR (>7.6%) or positive extranodal extension (ENE) had significantly worse clinical outcomes than patients with low-risk LNR (≤7.6%) or negative ENE [45]. In another of our previous studies, BMI was also found to be an independent prognostic factor. Compared to normoweight patients, overweight and obese patients had better clinical outcomes, while underweight status was associated with poor survival [10]. Furthermore, normoweight patients with cTNM stage IVA disease benefited from TPF induction chemotherapy followed by surgery and postoperative radiation, compared to surgery and postoperative radiation alone, in terms of OS and DMFS. In this study, the dNLR was found to be an independent prognostic factor for assessing the survival of OSCC patients, even after adjusting for other important variables. This finding is consistent with many other clinical trials that reported a significant relationship between low dNLR values and a good prognosis or favorable clinical or pathological responses [11,14,18,31,43,46,47].

In the present study, to account for the potential influence of TPF induction chemotherapy on inflammation biomarkers, hematology and survival data from the control group were used to form the ROC curve and calculate the AUC for the dNLR cutoff point. All four survival rates pointed to the same dNLR cutoff point of 1.555. This demonstrated that the dNLR can be an independent prognostic factor for the survival of OSCC patients based on multivariate analysis. There have been other reported dNLR cutoff points for different types of tumors. For instance, a dNLR cutoff point of 1.775 was found useful in predicting metastatic disease in testicular germ-cell tumors [47]. Another study on breast cancer reported a baseline dNLR cutoff point of less than 1.715 in predicting pathological complete response [46]. In patients treated with the immune checkpoint inhibitor pembrolizumab for non-small cell lung cancer, those with a dNLR cutoff < 2.6 had significantly higher objective response rates (ORRs), longer median progression-free survival, and higher numbers of tumor-associated CD8+, FOXP3+, PD-1+ immune cells, and PD-1+ CD8+ T cells [11]. In the Lung Immune Prognostic Index (LIPI), a dNLR cutoff of 3 was used to determine the prognosis of non-small cell carcinoma [14]. Various factors, such as tumor type, TNM stage, systemic inflammation, and population differences, contribute to these differing cutoff points. This may indicate the more malignant biological behavior of OSCC compared to other tumor types.

It was reported that a lower baseline dNLR level is associated with a higher number of tumor-associated immune cells [11]. In this study, a statistically significant correlation between the dNLR and cTNM stage (Table 1) was found in the control group, suggesting a mutual relationship where cTNM stage IVA disease often coincides with a high dNLR. Similar results have been reported for colorectal cancer, renal cell carcinoma, and gastric cancer [48,49,50], where TNM stage is positively related to dNLR. A low dNLR is associated with significantly increased tumor-associated CD8+, FOXP3+, and PD-1+ immune cells and favorable outcomes [11]. Stage III and stage IVA are all considered local advanced diseases in OSCC; thus, this difference was neglected in the following analysis.

A high baseline dNLR level indicates a relatively higher number of neutrophils. Neutrophils exhibit diversity, heterogeneity, and plasticity in cancer [51,52,53]. Different subgroups of neutrophils, each involved in distinct biological processes, have been reported [52,54]. In mouse and human lung cancer models, seven populations of neutrophils were identified, and CD40 agonist antibody treatment increased immune response by more than 10-fold in both N1a (Sellhi Ngphi) and N2 (Sellhi Cxcl10hi) neutrophil populations, characterized by high expression of interferon-stimulated genes (ISGs) [54]. This suggests that individuals with high baseline dNLR levels may lack the capacity for neutrophil differentiation into ISG-high expression subtypes, leading to an unfavorable prognosis. Furthermore, in the tumor immune microenvironment, tumors can modulate neutrophil extracellular traps (NETosis), causing NET-associated complications like thrombosis. NETosis captures tumors, promotes their growth, and leads to subsequent metastasis [55]. In breast cancer, the tumor-secreted protease cathepsin C promotes breast-to-lung metastasis by regulating the recruitment of neutrophils and the formation of neutrophil extracellular traps [56].

A high baseline dNLR level can also be due to low levels of leukocytes other than neutrophils, primarily lymphocytes. Lymphocytes play a crucial role in tumor immunity. However, the functional state of T lymphocytes is also critical to tumor immunity. T cell dysfunction in human cancer is associated with changes in T cell functionality rather than inactivity [57]. Although low dNLR levels predict a better prognosis in this study, the state of lymphocytes should still be considered.

There were some limitations to this study. Typically, patients with LAOSCC diagnosed as cTNM stage IVA have worse survival outcomes, which could lead to potential bias. Fortunately, this was not observed in the TPF induction chemotherapy group. This issue could be addressed in another clinical trial with a larger sample size to verify the possible connection between TNM stage and baseline dNLR in LAOSCC. Additionally, the retrospective nature of the study may include unnecessary confounding factors, and another clinical trial is needed to confirm whether the dNLR cutoff point is a suitable boundary for other survival rates or OSCC patients with different systemic conditions or TNM stages.

## 5. Conclusions

The dNLR can be considered an independent negative prognostic factor for the prognosis of OSCC patients. Patients with a baseline dNLR ≤ 1.555 have a better prognosis. Patients with stage III disease and a dNLR ≤ 1.555 can benefit from TPF induction chemotherapy, but further studies are needed to explain the biological association.

## Figures and Tables

**Table 1:** Demographic and clinical data of the OSCC patients.

		Total		Control Group						TPF Chemotherapy Induction					
				dNLR ≤ 1.555		dNLR > 1.555		t/χ^2^/Fisher Exact Test		dNLR ≤ 1.555		dNLR > 1.555		t/χ^2^/Fisher Exact Test	
		N	%	N	%	N	%		*p*	N	%	N	%		*p*
Age (years)															
	Average	55.4		54.8		56.1				56		56.2			
	Range	26, 75		26, 75		29, 74				32, 73		29, 74			
	<60	168	66	45	73	40	61	2.055	0.152	49	67	34	62	0.387	0.534
	≥60	88	34	17	27	26	39			24	33	21	38		
Gender															
	Female	77	30	20	32	20	30	0.057	0.812	21	29	16	29	0.002	0.968
	Male	179	70	42	68	46	70			52	71	39	71		
Smoking status															
	Negative	126	49	39	63	32	48	2.691	0.101	36	49	23	42	0.710	0.400
	Positive	130	51	23	37	34	52			37	51	32	58		
Alcohol status															
	Negative	98	38	42	68	40	61	0.707	0.400	45	62	31	56	0.363	0.547
	Positive	158	62	20	32	26	39			28	38	24	44		
Tumor site															
	Tongue	113	44	31	50	29	44	2.568	0.766	34	47	19	35	4.352	0.500
	Buccal	45	18	9	15	11	17			13	18	12	22		
	Gingiva	40	16	9	15	10	15			12	16	9	16		
	Floor of the mouth	30	12	6	10	12	18			6	8	6	11		
	Palate	18	7	3	5	3	5			5	7	7	13		
	Retromolar triangle	10	4	4	6	1	2			3	4	2	4		
BMI															
	Underweight	20	8	5	8	6	9	1.137	0.258	5	7	4	8	0.501	0.617
	Normal	132	52	34	55	39	59			35	49	24	45		
	Overweight	72	28	16	26	17	26			24	33	15	28		
	Obese	29	11	7	11	4	6			8	11	10	19		
cT stage															
	T1	9	4	0	0	2	3	1.637	0.749	4	5	3	5	0.911	0.937
	T2	57	22	12	19	13	20			20	27	12	22		
	T3	149	58	40	65	40	61			38	52	31	56		
	T4	41	16	10	16	11	17			11	15	9	16		
cN stage															
	N0	110	43	31	50	30	45	3.516	0.172	29	40	20	36	0.397	0.820
	N1	94	37	23	37	19	29			30	41	22	40		
	N2	52	20	8	13	17	26			14	19	13	24		
Clinical TNM stage															
	III	177	69	50	81	43	65	3.863	0.049	49	67	35	64	0.169	0.681
	IVa	79	31	12	19	23	35			24	33	20	36		

OSCC: oral squamous cell carcinoma; TPF: docetaxel, cisplatin, and 5-fluorouracil (5-FU); dNLR: derived neutrophil to lymphocyte ratio; BMI: body mass index.

**Table 2:** The 5-year survival outcomes of the patients with different treatments and different baseline dNLR levels.

	Control Group (N = 115)		Experimental Group (N = 109)	
	Low dNLR	High dNLR	Low dNLR	High dNLR
	(n = 56)	(n = 59)	(n = 63)	(n = 46)
Overall survival	69.4%	40.4%	77.4%	35.7%
Disease-free survival	57.8%	30.4%	74.0%	28.1%
Locoregional recurrence-free survival	62.9%	35.9%	74.0%	32.1%
Distant metastasis-free survival	67.7%	40.4%	80.8%	35.7%

TPF: docetaxel, cisplatin, and 5-fluorouracil (5-FU); dNLR: derived neutrophil to lymphocyte ratio.

**Table 3:** Univariate analysis of clinical prognostic factors for clinical outcomes in the control group (surgery and postoperative radiation).

Variable	OS			DFS			LRFS			DMFS		
	HR	95% CI	*p*	HR	95% CI	*p*	HR	95% CI	*p*	HR	95% CI	*p*
Sex (vs. Female)	0.965	0.568–1.642	0.897	0.736	0.452–1.199	0.219	0.744	0.453–1.222	0.242	0.985	0.580–1.676	0.957
Age (vs. < 60 years)	1.199	0.716–2.009	0.490	1.066	0.654–1.738	0.796	1.075	0.654–1.768	0.775	1.166	0.697–1.952	0.559
Smoking status (vs. non-smoker)	1.219	0.743–2.000	0.433	1.021	0.639–1.630	0.932	1.013	0.630–1.630	0.956	1.225	0.747–2.009	0.421
Alcohol status (vs. non-alcohol abuser)	1.385	0.807–2.376	0.237	1.441	0.867–2.394	0.158	1.339	0.799–2.245	0.286	1.432	0.834–2.456	0.193
BMI at diagnosis			0.005			0.009			0.007			0.008
Normal	Ref.			Ref.			Ref.			Ref.		
Underweight	2.145	1.065–4.321	0.033	1.915	0.961–3.814	0.065	2.106	1.053–4.210	0.035	2.067	1.027–4.162	0.042
Overweight	0.527	0.270–1.029	0.060	0.581	0.319–1.062	0.077	0.113	0.334–1.122	0.612	0.538	0.276–1.051	0.070
Obese	0.398	0.123–1.287	0.124	0.332	0.103–1.069	0.064	0.345	0.107–1.114	0.075	0.399	0.123–1.292	0.125
cTNM (vs. III)	1.993	1.184–3.354	0.009	1.792	1.088–2.952	0.022	1.939	1.173–3.207	0.010	1.603	1.127–3.190	0.016
dNLR	1.227	1.099–1.369	<0.001	1.186	1.063–1.324	0.002	1.200	1.073–1.341	0.001	1.218	1.091–1.361	<0.001
NLR	1.073	0.936–1.230	0.311	1.098	0.979–1.231	0.109	1.113	0.995–1.245	0.061	1.074	0.935–1.232	0.313
PLR	1.002	0.997–1.007	0.391	1.004	1.000–1.008	0.044	1.005	1.000–1.009	0.030	1.002	0.997–1.007	0.399
LMR	0.959	0.828–1.111	0.577	0.933	0.811–1.075	0.338	0.913	0.788–1.057	0.222	0.958	0.828–1.109	0.568
PIV	1.000	0.999–1.001	0.991	1.000	0.999–1.001	0.985	1.000	0.999–1.001	0.925	1.000	0.999–1.001	0.991

OS: overall survival; DFS: disease-free survival; LRFS: locoregional recurrence-free survival; DMFS: distant metastasis-free survival; HR: hazard ratio; BMI: body mass index; dNLR: derived neutrophil to lymphocyte ratio; NLR: neutrophil to lymphocyte ratio; PLR: platelet to lymphocyte ratio; LMR: lymphocyte to monocyte ratio; PIV: pan-immune inflammation value.

**Table 4:** Multivariate analysis of clinical prognostic factors for clinical outcomes in the control group (surgery and postoperative radiation).

Variable	OS			DFS			LRFS			DMFS		
	HR	95% CI	*p*	HR	95% CI	*p*	HR	95% CI	*p*	HR	95% CI	*p*
BMI at diagnosis			0.067			0.063			0.062			0.083
Normal	Ref.			Ref.			Ref.			Ref.		
Underweight	1.557	0.708–3.425	0.271	1.495	0.700–3.191	0.299	1.662	0.777–3.554	0.190	1.515	0.689–3.335	0.302
Overweight	0.510	0.995–1.268	0.061	0.574	0.313–1.050	0.071	0.605	0.329–1.111	0.105	0.523	0.267–1.024	0.059
Obese	0.477	0.146–1.557	0.220	0.385	0.118–1.250	0.112	0.400	0.123–1.301	0.128	0.475	0.146–1.552	0.218
cTNM (vs. III)	1.911	1.098–3.328	0.022	1.628	0.959–2.763	0.071	1.756	1.029–2.996	0.039	1.797	1.003–3.125	0.038
dNLR	1.154	1.018–1.309	0.025	1.123	1.001–1.282	0.050	1.134	1.002–1.283	0.047	1.146	1.010–1.300	0.035
PLR	1.002	0.996–1.008	0.505	1.005	0.999–1.011	0.092	1.005	0.999–1.010	0.107	1.002	0.996–1.008	0.498

OS: overall survival; DFS: disease-free survival; LRFS: locoregional recurrence-free survival; DMFS: distant metastasis-free survival; HR: hazard ratio; BMI: body mass index; dNLR: derived neutrophil to lymphocyte ratio; PLR: platelet to lymphocyte ratio.

**Table 5:** Univariate analysis of clinical prognostic factors for clinical outcomes in the experimental group (TPF induction chemotherapy, surgery, and postoperative radiation).

Variable	OS			DFS			LRFS			DMFS		
	HR	95% CI	*p*	HR	95% CI	*p*	HR	95% CI	*p*	HR	95% CI	*p*
Sex (vs. Female)	1.212	0.657–2.237	0.539	1.219	0.687–2.160	0.499	1.189	0.670–2.111	0.555	1.207	0.654–2.228	0.548
Age (vs. < 60 years)	1.193	0.682–2.087	0.535	1.186	0.704–1.996	0.521	1.213	0.719–2.047	0.470	1.183	0.676–2.068	0.556
Smoking status (vs. non-smoker)	1.366	0.785–2.378	0.270	1.266	0.757–2.116	0.369	1.221	0.728–2.048	0.448	1.374	0.789–2.393	0.261
Alcohol status (vs. non-alcohol abuser)	1.144	0.661–1.978	0.631	1.175	0.706–1.955	0.534	1.124	0.672–1.880	0.656	1.135	0.656–1.963	0.650
BMI at diagnosis			0.550			0.504			0.511			0.567
Normal	Ref.			Ref.			Ref.			Ref.		
Underweight	1.549	0.593–4.051	0.372	1.256	0.488–3.236	0.637	1.314	0.509–3.393	0.573	1.564	0.598–4.088	0.362
Overweight	0.743	0.380–1.453	0.386	0.659	0.351–1.240	0.196	0.676	0.358–1.276	0.227	0.757	0.387–1.480	0.415
Obese	1.065	0.480–2.361	0.877	1.019	0.485–2.140	0.961	1.054	0.500–2.220	0.890	1.065	0.480–2.362	0.876
cTNM (vs. III)	1.588	1.015–2.755	0.022	2.461	1.398–4.331	0.002	2.493	1.413–4.399	0.009	1.603	1.060–3.500	0.031
dNLR	1.154	1.035–1.285	0.010	1.141	1.029–1.266	0.013	1.139	1.024–1.268	0.016	1.152	1.034–1.282	0.010
NLR	1.286	0.953–1.737	0.100	1.326	1.015–1.731	0.038	1.315	1.005–1.722	0.046	1.309	0.967–1.772	0.082
PLR	1.009	1.000–1.017	0.051	1.009	1.002–1.017	0.017	1.008	1.000–1.016	0.040	1.009	1.000–1.017	0.054
LMR	1.092	0.921–1.295	0.312	1.038	0.879–1.225	0.662	1.039	0.879–1.229	0.652	1.090	0.920–1.293	0.320
PIV	1.000	0.999–1.001	0.991	1.000	0.999–1.001	0.985	1.001	1.000–1.002	0.116	1.000	0.999–1.001	0.991

TPF: docetaxel, cisplatin, and 5-fluorouracil (5-FU); OS: overall survival; DFS: disease-free survival; LRFS: locoregional recurrence-free survival; DMFS: distant metastasis-free survival; HR: hazard ratio; BMI: body mass index; dNLR: derived neutrophil to lymphocyte ratio.

**Table 6:** Multivariate analysis of clinical prognostic factors for clinical outcomes in the experimental group (TPF induction chemotherapy, surgery, and postoperative radiation).

Variable	OS			DFS			LRFS			DMFS		
	HR	95% CI	*p*	HR	95% CI	*p*	HR	95% CI	*p*	HR	95% CI	*p*
cTNM (vs. III)	1.924	1.057–3.499	0.032	1.926	1.154–3.215	0.012	1.991	1.188–3.337	0.024	1.926	1.014–2.782	0.033
dNLR	1.160	1.031, 1.276	0.011	1.239	1.019, 1.250	0.021	1.125	1.014, 1.249	0.027	1.145	1.030, 1.272	0.012
PLR	1.008	0.998–1.019	0.125	1.009	1.000–1.019	0.052	1.008	0.998–1.017	0.145	1.008	0.997–1.018	0.154

TPF: docetaxel, cisplatin, and 5-fluorouracil (5-FU); OS: overall survival; DFS: disease-free survival; LRFS: locoregional recurrence-free survival; DMFS: distant metastasis-free survival; HR: hazard ratio; dNLR: derived neutrophil to lymphocyte ratio; PLR: platelet to lymphocyte ratio.

## Data Availability

The data presented in this study are available in this article and Supplementary Material.

## Supplementary Materials

The following supporting information can be downloaded at: https://www.mdpi.com/article/10.3390/cancers16152707/s1, Figure S1. Survival analysis of (A) overall survival, (B) disease-free survival, (C) locoregional recurrence-free survival, and (D) distant metastasis-free survival between the control group and the experimental group. TPF, docetaxel, cisplatin, and 5-fluorouracil (5-FU). Figure S2. Survival analysis of (A) overall survival, (B) disease-free survival, (C) locoregional recurrence-free survival, and (D) distant metastasis-free survival in the low-dNLR patient group. Survival analysis of (E) overall survival, (F) disease-free survival, (G) locoregional recurrence-free survival, and (H) distant metastasis-free survival in the high-dNLR patient group. dNLR, derived neutrophils to lymphocyte ratio; TPF, docetaxel, cisplatin, and 5-fluorouracil (5-FU). Figure S3. Survival analysis of (A) overall survival, (B) disease-free survival, (C) locoregional recurrence-free survival, and (D) distant metastasis-free survival in the patient group with cTNM stage III disease. Survival analysis of (E) overall survival, (F) disease-free survival, (G) locoregional recurrence-free survival, and (H) distant metastasis-free survival in the patients with cTNM stage IVA disease. dNLR, derived neutrophils to lymphocyte ratio; TPF, docetaxel, cisplatin, and 5-fluorouracil (5-FU). Figure S4. Schoenfeld residuals for proportinoal hazards derived neutrophil to lymphocyte ratio in (A) overall survival, (B) disease-free survival, (C) locoregional recurrence-free survival, and (D) distant metastasis-free survival and platelet to lymphocyte ratio in (E) overall survival, (F) disease-free survival, (G) locoregional recurrence-free survival, and (H) distant metastasis-free survival. Table S1. Sensitivity analysis of overall survival in patients treated with surgery and postoperative radiation. Table S2. Sensitivity analysis of disease-free survival in patients treated with surgery and postoperative radiation. Table S3. Sensitivity analysis of locoregional recurrence-free survival in patients treated with surgery and postoperative radiation. Table S4. Sensitivity analysis of distant metastasis-free survival in patients treated with surgery and postoperative radiation. Table S5. Model fit using Akaike Information Criterion (AIC).
