# Supplementary figures and images for: Derived Neutrophils to Lymphocyte Ratio Predicts Survival Benefit from TPF Induction Chemotherapy in Local Advanced Oral Squamous Cellular Carcinoma
Source: Cancers (Basel). 2024 Jul 30;16(15):2707. doi: 10.3390/cancers16152707 (PMC11311474; doi:10.3390/cancers16152707)

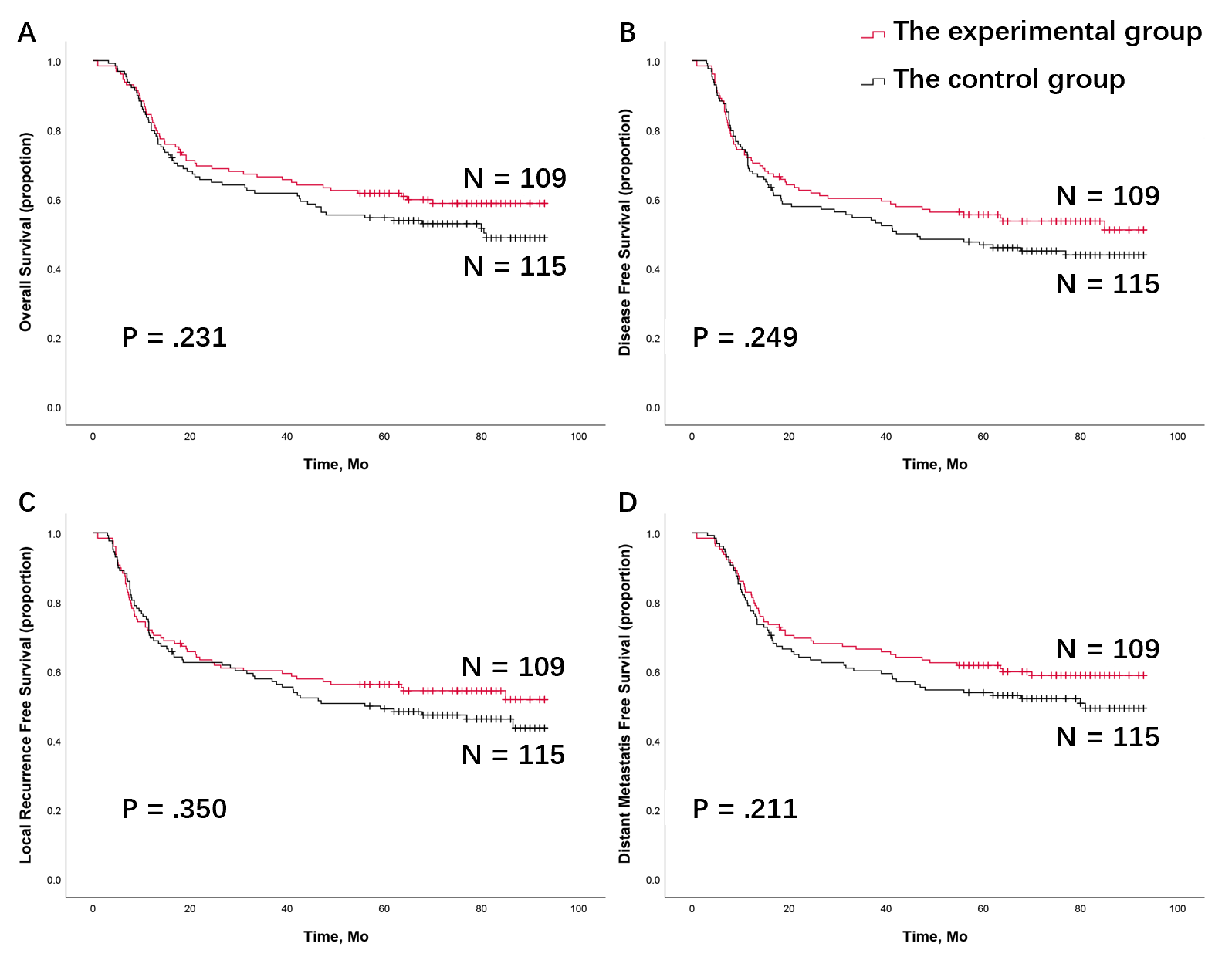

Supplement: Supplementary file 1 [file cancers-16-02707-s001.zip › Supplementary figure S1.tif]

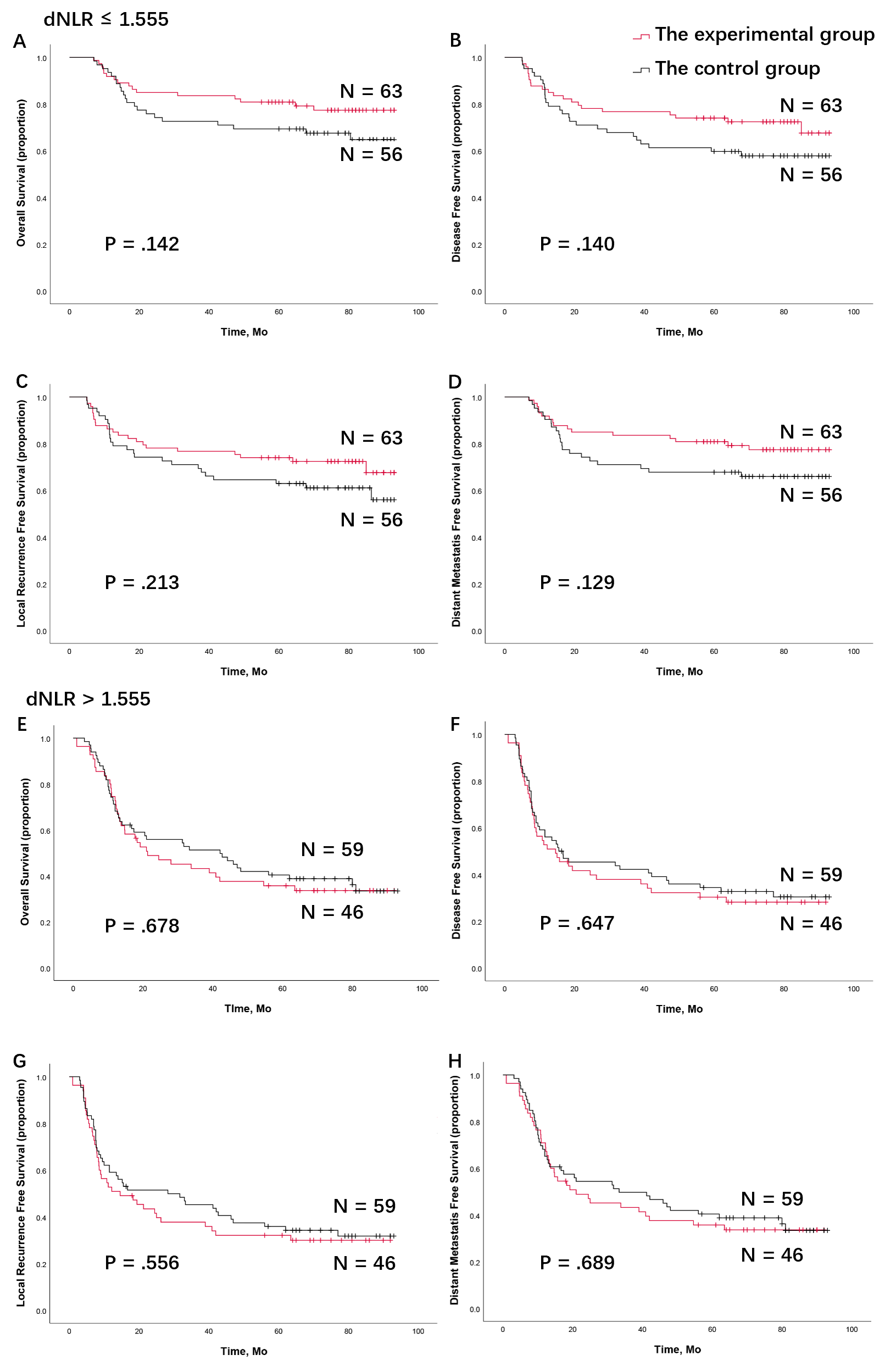

Supplement: Supplementary file 1 [file cancers-16-02707-s001.zip › Supplementary figure S2.tif]

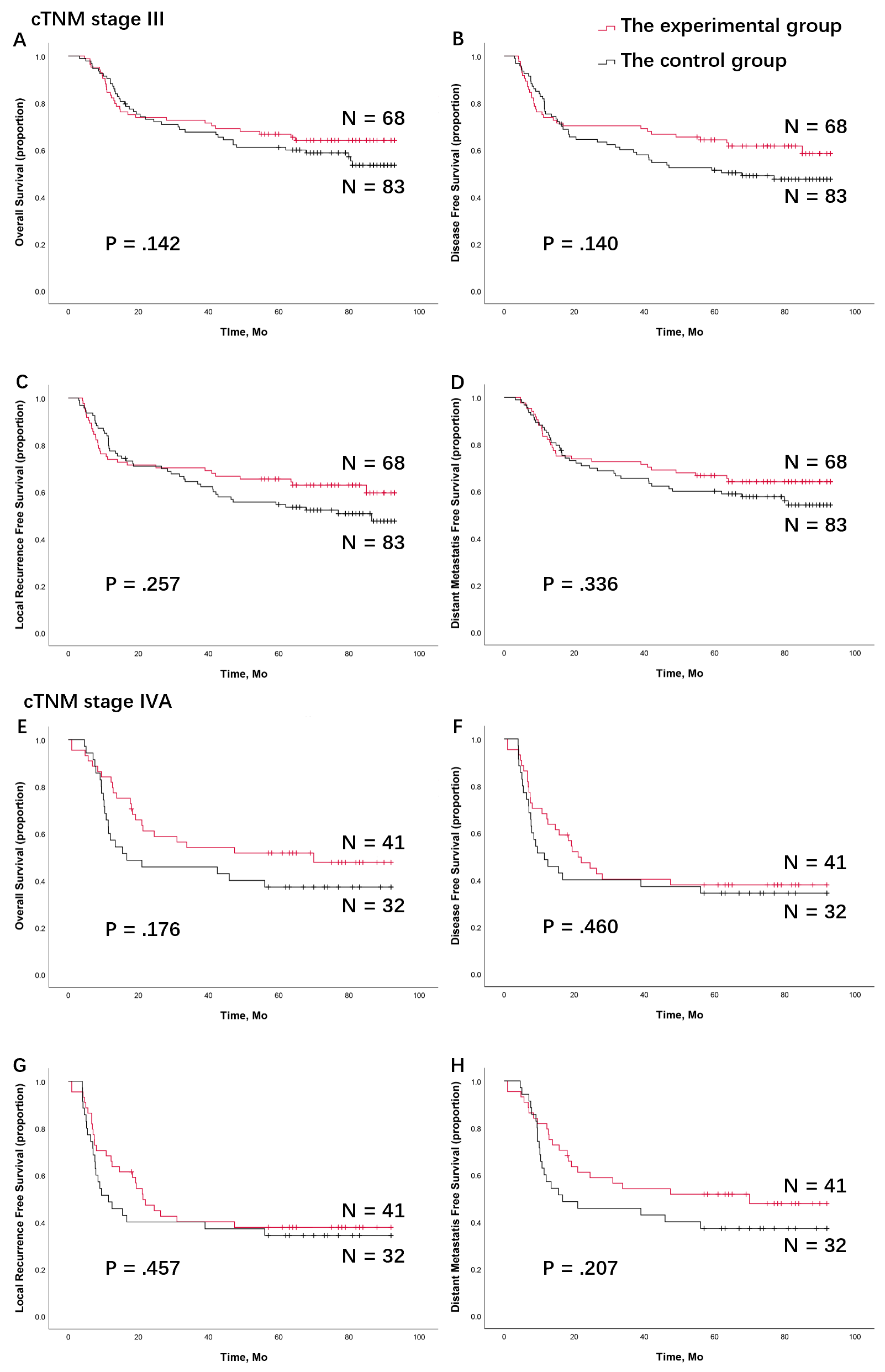

Supplement: Supplementary file 1 [file cancers-16-02707-s001.zip › Supplementary figure S3.tif]

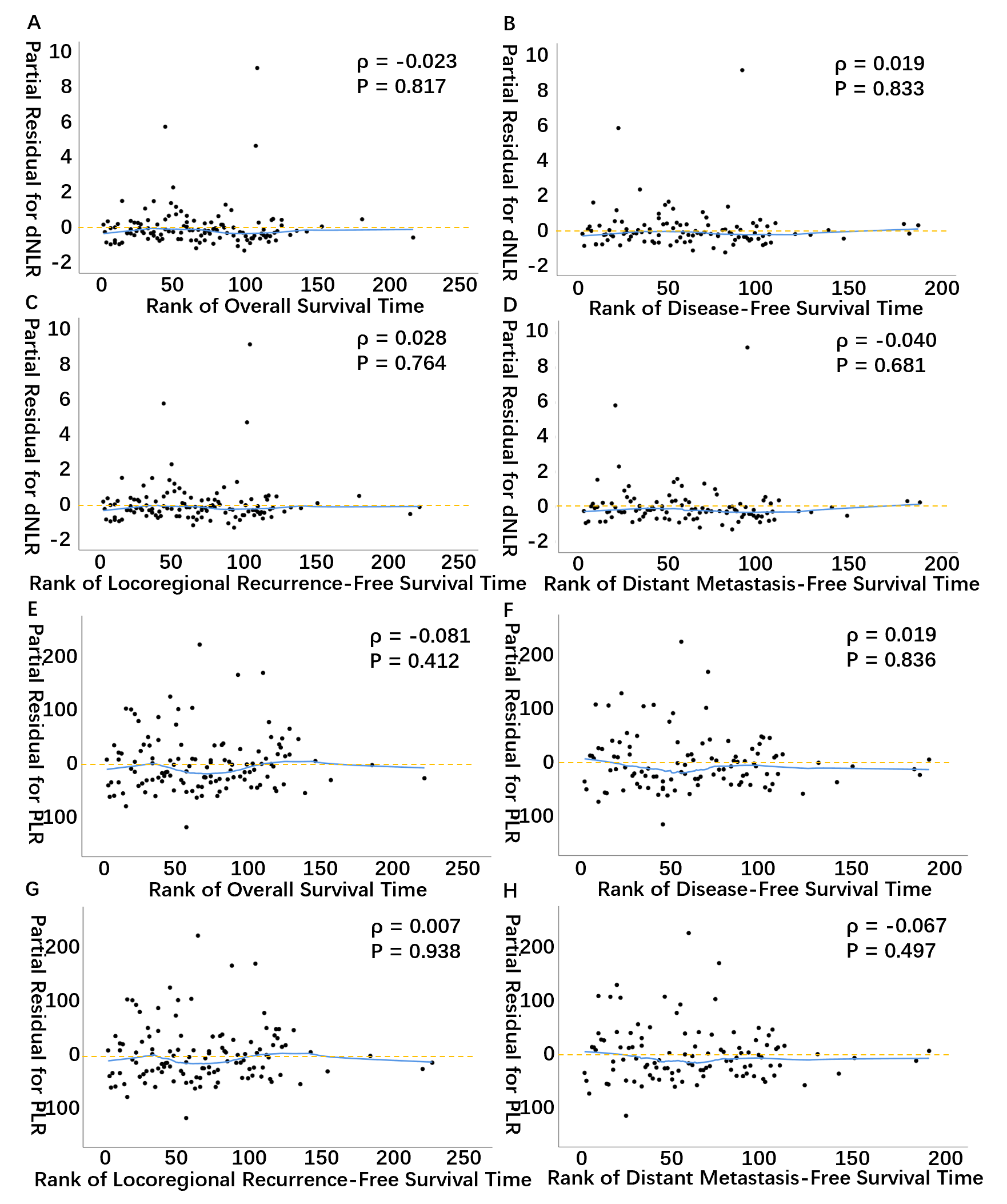

Supplement: Supplementary file 1 [file cancers-16-02707-s001.zip › Supplementary figure S4.tif]
